# *In vivo* Regeneration of Mineralized Bone Tissue in Anisotropic Biomimetic Sponges

**DOI:** 10.3389/fbioe.2020.00587

**Published:** 2020-07-07

**Authors:** Janeth Serrano-Bello, Iriczalli Cruz-Maya, Fernando Suaste-Olmos, Patricia González-Alva, Rosaria Altobelli, Luigi Ambrosio, Luis Alberto Medina, Vincenzo Guarino, Marco Antonio Alvarez-Perez

**Affiliations:** ^1^Tissue Bioengineering Laboratory, Postgraduate Studies and Research Division, Faculty of Dentistry, National Autonomous University of Mexico, Mexico City, Mexico; ^2^Institute of Polymers, Composites, and Biomaterials, National Research Council of Italy, Naples, Italy; ^3^Instituto de Fisiología Celular, Universidad Nacional Autónoma de México, Mexico City, Mexico; ^4^Institute of Composite and Biomedical Materials, National Research Council of Italy, Naples, Italy; ^5^Instituto de Física, Universidad Nacional Autónoma de México, Mexico City, Mexico; ^6^Unidad de Investigación Biomédica en Cáncer, Instituto Nacional de Cancerología/Universidad Nacional Autónoma de México, Mexico City, Mexico

**Keywords:** alginate, hydroxyapatite, anisotropic structure, microporous sponges, hard tissues, *in vivo* models

## Abstract

In the last two decades, alginate scaffolds have been variously studied as extracellular matrix analogs for tissue engineering. However, relevant evidence is still lacking concerning their ability to mimic the microenvironment of hierarchical tissues such as bone. Hence, an increasing amount of attention has recently been devoted to the fabrication of macro/microporous sponges with pore anisotropy able to more accurately replicate the cell niche structure as a trigger for bioactive functionalities. This paper presents an *in vivo* study of alginate sponges with anisotropic microporous domains (MAS) formed by ionic crosslinking in the presence of different fractions (30 or 50% v) of hydroxyapatite (HA). In comparison with unloaded sponges (MAS0), we demonstrated that HA confers peculiar physical and biological properties to the sponge, depending upon the inorganic fraction used, enabling the sponge to bio-mimetically support the regeneration of newly formed bone. Scanning electron microscopy analysis showed a preferential orientation of pores, ascribable to the physical constraints exerted by HA particles during the pore network formation. Energy dispersive spectroscopy (EDS) and X-Ray diffraction (XRD) confirmed a chemical affinity of HA with the native mineral phase of the bone. *In vitro* studies via WST-1 assay showed good adhesion and proliferation of human Dental Pulp-Mesenchymal Stem Cells (hDP-MSC) that increased in the presence of the bioactive HA signals. Moreover, *in vivo* studies via micro-CT and histological analyses of a bone model (e.g., a rat calvaria defect) confirmed that the maximum osteogenic response after 90 days was achieved with MAS30, which supported good regeneration of the calvaria defect without any evidence of inflammatory reaction. Hence, all of the results suggested that MAS is a promising scaffold for supporting the regeneration of hard tissues in different body compartments.

## Introduction

The ability to reproduce the complex hierarchal organization of a tissue architecture still represents one of the greatest challenges for the regeneration and repair of a broad range of damaged anisotropic tissues (Perez et al., 2018). It is well known that the absence of a porous hierarchical structure may represent a strong limitation for cells in terms of directional migration and spatial organization, consequently compromising morpho-functional integration (Zhu et al., 2019). For this reason, the extracellular matrix (ECM) of most human tissues naturally exhibits heterogeneous anisotropic domains that are characterized by the presence of pore gradients with some peculiar differences as a function of the specific bone tissue (Kang et al., 2018).

In the last two decades, the efficient use of anisotropic porous scaffolds has been largely validated in bone surgery, due to the opportunity to impart instructive functions to cells – by optimizing specific features of the materials (i.e., porosity, roughness, stiffness, biodegradation, fluid, and molecular transport) – able to synchronously guide the regeneration process, while the porous scaffold disappears in the local microenvironment (Guarino et al., 2012b). In this view, the gold standard is currently represented by composite scaffolds obtained by the combination of polymer and ceramic biomaterials, sagely processed to provide a well-defined pattern of physical and biochemical signals able to trigger the basic activities of progenitor cells toward a more efficient proliferation and cell differentiation (Funda et al., 2020). In this context, hydroxyapatite (HA), a calcium phosphate compound electively used in the pure formulation for bone repair (Mohd Pu’ad et al., 2019) due to the excellent physicochemical properties it derives from its monoclinic (P2_1_/b) or hexagonal (P6_3_/m) crystalline structure, and with a Ca/P molar ratio of 1.67 similar to human bone (Dong-Hyun et al., 2015), has been preferentially used in combination with biodegradable polymers [i.e., polylactic acid (PLA) and polycaprolactone (PCL)] for tissue engineering (Guarino et al., 2012a). Indeed, bioresorbable properties coupled with proved osteoconductive response make HA an ideal candidate for preventing any local or systemic toxicity, inflammation, or foreign response in host tissues (Guarino et al., 2014). Moreover, the use of formulations doped with specific chemical elements (i.e., Zn, and Mg) can promote direct chemical bonds with the adjacent natural bone tissue by assuring higher stability of crystals into the body fluids as the osteointegration mechanism goes on (Veronesi et al., 2015).

Taking into consideration the natural composition of the natural bone matrix – made of biologically recognized ceramics (i.e., hydroxyapatite) and bioactive proteins (i.e., collagen) – current interest is gradually addressing the design of scaffolds from naturally available sources. These scaffolds, based on polysaccharides (Dinoro et al., 2019), proteins (Yadav and Srivastava, 2019), and polyalkenoates (Elmowafy et al., 2019), show several biochemical affinities with biological macromolecules naturally present into the ECM and are, therefore, able to support a more efficient control of molecular transport, with relevant benefits for cell signaling at *in vivo* regeneration (Li et al., 2014; Ki et al., 2019). Among them, alginates represent one of the most interesting choices, being certified by the Food and Drug Administration (FDA) for tissue engineering due to their chemical similarity to ECM components, which ensures their excellent biocompatibility, immunological non-responsiveness (Li et al., 2015), and *in vivo* stability (Guarino et al., 2015). Alginate is an anionic polysaccharide derived from seaweed. It is composed of blocks of 1,4-linked β-D-mannuronate (M residues) and 1,4-linked α-L-guluronate (G residues) that can interact by different chemical/physical crosslinking methods [i.e., ionic interaction, free radical polymerization, or click reaction (Sun and Tan, 2013)] to form stable hydrogels with controlled porosity as a function of the reaction triggers (i.e., Ca^2+^, Ba^2+^, Mg^2+^, Fe^2+^, Al^3+^, La^3+^, Pr^3+^, and Nd^3+^; Zhang et al., 2017).

Accordingly, several works have investigated the biological validation of alginate scaffolds in combination with calcium phosphates in the form of HA (Suárez-González et al., 2010) or bioactive glasses (Haider et al., 2020) to develop bioactive scaffolds suitable for mimicking the mineralized ECM to promote *in vitro* cell differentiation or *in vivo* cell response in an osteogenic way (Jin et al., 2012; Singh et al., 2014). However, no studies have yet investigated the biological response in the presence of bioactive alginate scaffolds with an anisotropic structure.

Herein, we have proposed the fabrication of microporous alginate sponges (MAS) loaded with HA at loadings of 30% (MAS30) and 50% (MAS50) via ionic crosslinking. It was demonstrated that the addition of HA rigid phases influences the mechanism of phase transition during the sponge fabrication, thus inducing the formation of macro-domains with anisotropic properties – i.e., preferential orientation of pores into a single domain. In this study, the effect of this peculiar scaffold architecture on hDP-MSC behavior will be investigated, and long-term *in vivo* application on a bone defect model in calvaria of rats will be validated for mineralized tissue regeneration.

## Materials and Methods

### Morphological Characterization

Microporous alginate sponges were prepared as reported elsewhere (Guarino et al., 2008). A brief description of the process is schematized in Supplementary Figure S1. Microporous alginate sponges morphology was investigated with scanning electron microscopy (SEM) in secondary electron mode using a field emission microscope (FESEM Quanta 200, FEI, Netherlands). Specimens were fractured in liquid nitrogen by using a razor blade along preferred directions parallel and perpendicular to the surface. The resulting transverse and longitudinal sections were gold-coated under a vacuum using an automatic coating sputter set at 15 mA for about 20 min (Emiscope SC500, Italy). Moreover, X-ray energy dispersive spectroscopy (EDS, Genesis 2000i) was used for qualitative mapping and quantitative analysis of chemical elements composing inorganic phases into the composite sponges.

### *In vitro* Biocompatibility of Microporous Alginate Sponges

To establish the effect of the peculiar MAS scaffold architecture on biocompatibility, human dental pulp stem cells (hDP-MSC) were used. Cells were maintained in α-MEM supplemented with 10% FBS and a solution of antibiotics (penicillin 100 IU/mL and streptomycin 100 μg/mL) at 37°C in a humidified atmosphere with 5% CO_2_. Cellular biocompatibility was evaluated by analyzing two important processes: (1) cell adhesion and (2) cell viability of hDP-MSC by WST-1 assay. Briefly, cells were seeded at 1 × 10^4^ cells/mL density in triplicate onto MAS0, MAS30, and MAS50. Cell adhesion was analyzed after 4 and 24 h of cell culture, and cell viability was evaluated after 2, 4, 6, and 8 days of culture. After the prescribed time period, substrates were rinsed three times with PBS to remove non-adherent cells and then incubated with 400 μL fresh culture medium containing 40 μL of the WST-1 solution for 4 h at 37°C. After the incubation time, 200 μL of the supernatant was placed in a 96-well plate, and the optical absorption was quantified by spectrophotometry at 450 nm with a plate reader (ChroMate, Awareness Technology, Palm City, FL, United States). Sponges without any presence of HA were used as control, and during the experimental viability time, the culture medium was changed every two days.

### *In vivo* Studies of the Osteogenic Capacity of Microporous Alginate Sponges

#### Animal Model

To evaluate the osteogenic capacity potential of MAS0, MAS30, and MAS50, a calvaria rat model was used for preliminary *in vivo* studies. The animals used for the animal model were male, young-adults *Wistar* rats weighing 250 g. They were kept under laboratory conditions at a temperature of 22°C, with a photoperiod of 12 h and relative humidity at 50%, and with food and water *ad libitum*. All rats were sedated and tranquilized with ketamine (80 mg/kg) and Xylazine (10 mg/kg), intramuscularly, and then the calvaria surgical site was shaved, followed by routine antisepsis. Briefly, a 3-cm linear incision was made through the skin and the shell of the periosteum to expose the cranial vertex. The critical defect was created by using a trephine with a diameter of 9 mm mounted on a surgical engine at 4,000 rpm. A defect of standardized size was created on the middle portion of the frontal bone by using a chisel to cleave the bone fragment while irrigating with sterile phosphate buffer (PBS). This was done carefully to avoid damaging the dura, and the site was rinsed with sterile PBS to remove debris. Subsequently, four groups were randomly formed as follows: (1) MAS0, (2) MAS30, (3) MAS50, and (4) without the presence of any scaffold (NT). After that, Satin S-100 was applied to fix the scaffold to the defect and also to prevent the remaining periosteum from invading the defect area. The surgical site in all animal groups was closed with continuous 4-0 polyglycolic acid suture. Finally, the external surgical wound was cleaned with sterile PBS. All groups were clinically evaluated during the study period, considering the Grimace scale, changes in behavior, fur, and weight. The study was performed following the Mexican Legislation (NOM-062-ZOO-1999) and fulfilled all legal requirements of the Internal Committee for the Care and Use of Laboratory Animals of the Faculty of Medicine, UNAM, with approval number 027-CIC-2019. At the end of the *in vivo* studies, the animals were euthanized with carbon dioxide; the samples were fixed and processed for histological evaluation, obtaining serial sections 5 μm thick and stained with hematoxylin and eosin (H&E).

#### Bone Assessment by μCT Images

*In vivo* studies of the rat calvaria were conducted under inhaled anesthesia (2% isoflurane/oxygen) at 8, 30, 60, and 90 days after surgery. Images were obtained with the micro-CT unit of an Albira small animal microPET/SPECT/CT imaging system (Bruker), using settings of a 0.4 mA current, 35 kV voltage, and a total of 1,000 projections over 360°. The field of view was located in the defect zone (calvaria). Image reconstruction was performed using the filtered back-projection algorithm (FBP) with Albira Reconstructor software, resulting in a 558 × 558 × 516 image matrix with voxel size 125 μm^3^, for a total height of 64.5 mm. OsiriX MD software (Pixmeo SARL, 2019) was used for processing and visualization of 2D and 3D images.

The bone mineral density (BMD) was calculated from attenuation values using a linear model (density calibration) obtained from scans of a HA phantom (MicroMouse Phantom 090 CIRS, United States), following a procedure recommended by the manufacturer of the Albira scanner (Sasser et al., 2012). The BMD values were normalized to bone tissue adjacent to the defect and used as an indicator of the quality of regenerated bone in reference to healthy tissue. To evaluate the quantity of bone regeneration, 3D images were registering at different time points to determine the precise locations of bone formation or resorption. Briefly, for each sample, a region of interest (ROI), represented by the area of regenerated bone at the defect site, was manually drawn in each of the 280 cuts obtained from the micro-CT images. Then, to determine its volume, the results were normalized by the total volume of defect size. Only tomographic transverse views were used for this calculation.

#### Histological Analysis

The scaffolds and the surrounding tissue were retrieved immediately after euthanasia and prepared for histological evaluation. In brief, tissue samples were thoroughly washed and fixed in 10% formalin for 24 h. Subsequently, the samples were demineralized in Evans and Krajian solution. Finally, samples were dehydrated in a series of alcohol solutions ranging from 50 to 100% and embedded in paraffin blocks. Embedded blocks were cut into serial sections (∼150 μm) and stained with H&E. Histological samples were qualitatively evaluated at various magnifications.

#### Statistical Analysis

Cell biocompatibility data are presented as mean ± standard deviation (*n* = 3). In the animal model, twenty-four male Wistar rats were used and randomly placed in four groups of six rats each. Data regarding the groups are presented as mean ± standard deviation (*n* = 6). The sample size corresponds to the value of the standard deviation, with *Z*α (*Z* of alpha) referring to the type I error (confidence level α = 0.05 corresponding to a value of *Z* = 1.96) and *Z*β (beta *Z*) with a power of 80% (value of *Z* = 0.84). Inter-group analysis of variance (ANOVA) was performed using the SPSS statistical analysis package (ver. 20) followed by *post hoc* test (Bonferroni) to make multiple comparisons between groups. A value of *p* < 0.05 was considered to determine statistically significant differences.

## Results and Discussion

In bone surgery, the regeneration of a large bone defect after tumor isolation, trauma, or infection still represents a tremendously relevant clinical issue. To date, the use of osteoconductive materials such as HA embedded in porous polymer matrices had been identified as the most valid strategy for creating ECM analogs that replicate the morphological and/or chemical signals of the bone microenvironment and trigger *in vitro* functions of cells, i.e., migration, proliferation, and differentiation from progenitor cells (Kazem-Arki et al., 2018). In the last few years, several efforts have been addressed to optimizing easy-to-use processes with the required flexibility/versatility to mix different organic and inorganic biomaterials and generate porous networks with controlled porosity features at the micro-/submicro-scale. In this view, it has been demonstrated that low viscous sodium alginate (SA) can promote the formation of fully interconnected porous sponges with average pore sizes of around 100 μm (Supplementary Figure S1) that are suitable for supporting cell adhesion and proliferation (Thein-Han and Misra, 2009), as confirmed by preliminary studies. However, the ability to generate anisotropic pore patterns still remains one of the crucial points to really mimic the complex architecture of bony ECM because it allows morphological signals to be imparted to cells that are able to orchestrate – spatially and temporally – the regeneration and repair of the mineralized tissue (Ueon et al., 2010).

**FIGURE 1:**
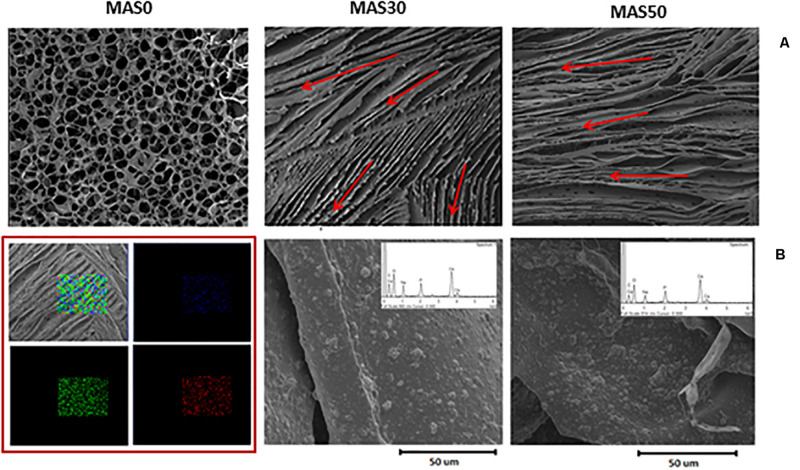
Pore morphology of alginate porous sponges as a function of the HA volume fraction. **(A)** SEM images of sponge cross-sections (scale bar: 1 mm). Pores originated by the development of crystal domains with preferential orientation (see red arrows). This was determined by the presence of rigid HA phases that constrain the formation of pores to make them anisotropic. **(B)** SEM images of the pore surface to evaluate HA distribution. In the red square, EDS analysis and mapping for MAS30: the results confirmed the homogeneous elemental distribution along a portion of the surface -carbon (green), calcium (red), and phosphorus (blue).

Herein, a consolidated and straightforward methodology was proposed to fabricate porous alginate sponges in agreement with previous studies (Haugh et al., 2010). In particular, the ion gelation mechanism was properly particularized in order to obtain a peculiar anisotropy of the three-dimensional pore network, mainly imparted by the presence of HA particles that stabilize the pore surfaces during the phase separation process. This was highlighted by the qualitative morphological study of alginate sponges with different HA volume fractions, MAS0, MAS30, and MAS50, respectively, which is extremely useful for investigating pore morphological features (Figure 1).

Certain HA fractions – 30 or 50%, respectively – were selected in order to partially mimic the natural composition of bone – i.e., the 30% organic matrix represented by type I collagen and 60% mineral phase, mainly HA crystals (Kolb and Bussard, 2019). In Figure 1A, SEM images of MAS30 and MAS50 clearly show the formation of macro-domains (see red arrows) in which the pores are organized in canaliform structures characterized by unidirectional orientation. This peculiar morphology of pores is strictly related to the preparation method used, which was based on the use of insulating molds and is able to trigger thermal flow gradients locally that give raise to the formation of oriented pores since the growth of ice crystals is confined into the single domain. Indeed, the growth of the ice crystals during the freeze-drying process follows the direction of thermal flow, and consequently, causes the pores to all be oriented in the same direction. This peculiar effect is strictly related to the presence of HA particles that – in consideration of their physical (e.g., rigidity) properties and geometry – offer a physical constraint to the growth of ice crystals. This effect is coupled with a general reduction in the pore size and the degree of interconnection as the HA content increases.

Accordingly, Figure 1B shows the pores at a higher magnification, revealing a homogeneous distribution of HA along the pore surfaces, with a tendency to form clusters at higher HA fractions. In the case of MAS30, this result was corroborated by the mapping of chemical elements via EDS analysis, which confirms the presence of apatites with a Ca/P ratio close to those of the native bone, with relevant benefits in terms of being an osteogenic interface for cells. More interestingly, macroscopically, MAS30 and MAS50 show a peculiar 3D lamellar architecture ascribable to the self-arrangement of pores with preferential orientations in macro-sized domains. This confers a peculiar anisotropic architecture to the sponges that positively influences the *in vivo* growth of the mineralized bone, in agreement with previous studies on similar alginate sponges with a controlled pore architecture (McMahon et al., 2013; Zhang et al., 2017; Kolb and Bussard, 2019).

**FIGURE 2:**
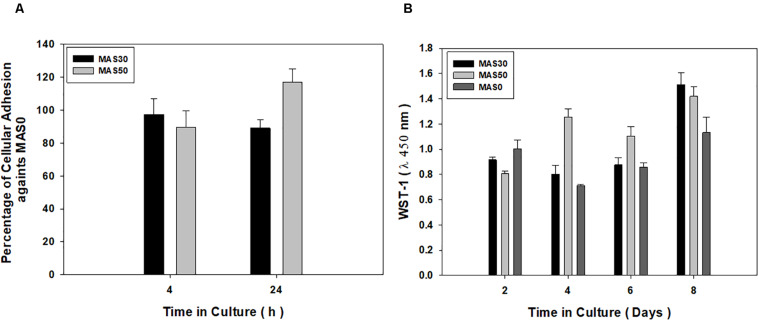
*In vitro* biological response of hDP-MSC to MAS0, MAS30, and MAS50. **(A)** Cell adhesion at 4 and 24 h and **(B)** cell viability after 2, 4, 6, and 8 days of cell culture.

Sponges were preliminarily investigated *in vitro* to evaluate the contribution of HA particles to adhesion and proliferation of hDP-MSC (Figure 2). The hDP-MSC cells adhered onto MAS pore surface scaffolds, independently of the HA content, as confirmed by there being no statistically significant differences after 4 and 24 h (Figure 2A). Cell viability was investigated at 2, 4, 6, and 8 days after seeding the hDP-MSC using WST-1 assay (Figure 2B). No difference in cell viability was detected in the cases of MAS30 and MAS50 at 2 days of cell culture in comparison with MAS0, which was used as a control. Contrariwise, an evident increase in cell viability was recognized after 4, 6, and 8 days in comparison with the control. Accordingly, viability assay demonstrated that all the sponge scaffolds are non-cytotoxic, also confirming the higher biocompatibility of MAS30 and MAS50 with respect to MAS0.

**FIGURE 3:**
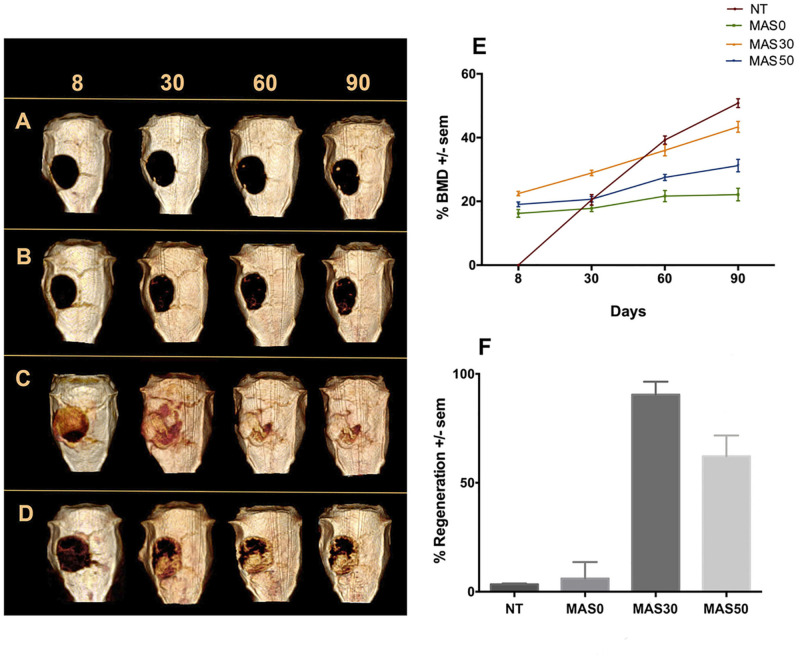
**(A)** Sequence of micro-CT images of sponges after 8, 30, 60, and 90 days in the *in vivo* calvaria defect model. **(A)** No treatment (NT), **(B)** MAS0, **(C)** MAS30, and **(D)** MAS50. **(E)** Measurement of bone mineral density (BMD) after 8, 30, 60, and 90 days in the calvaria defect model. **(F)** Percentage of *in vivo* total bone regeneration after 90 days in the calvaria defect model.

The capability to stimulate the recapitulation of mineralized ECM *in vivo* by the precipitation of mineral deposit calcification was explored for all of the samples via the rat calvaria defect model (Spicer et al., 2012). The different groups (MAS0, MAS30, MAS50, and the NT control group) were analyzed microtomographically at 8, 30, 60, and 90 days after surgery, and sequences of representative photographs of the groups at different time points were examined (Figures 3A–D). The NT group has a minimal region of regeneration in the defect area, i.e., the defect was still visible after 30 days, which increased in density but not in size at 60 and 90 days (Figure 3A). In the case of MAS0, 30 days after surgery, small trabeculae with variable density were recognized, mainly at the edges of the defect, and the response increased after 90 days (Figure 3B). The MAS30 group exhibited positive behavior since, from 8 days, the adaptation of the sponge to the defect area can be clearly observed. At 30 days, areas of hyperdense tissue deposited around the defect could be distinguished. The border center had a more significant amount of hyperdense tissue, which decreased in the upper and lower parts of the defect. At 60 and 90 days, similar behavior could be observed on the mineral tissue deposit; however, the density of the area was enhanced (Figure 3C). The MAS50 group was observed after 8 days to show correct sealing of the sponge edges to the defect, coupled with a limited mineral deposit. Moreover, after 30 to 90 days, an increased amount of mineral tissue had been deposited, with areas of variable density (Figure 3D).

The degree of mineralization in the defect area was evaluated by the percentage of BMD of the neo-formed tissue around the healthy adjacent bone at different periods of time of 8, 30, 60, and 90 days (Figure 3E). In the NT control group, the BMD was only quantified after 30 days, due to the limited size of the regenerated tissue (only at the edges of the defect). After 8 days, the BMD measured in the cases of MAS0 and MAS50 was comparable to the values measured after 30 days. At 30 days, it was equal to 20.47 ± 1.64% for the control, 17.78 ± 0.97% for MAS0, and 20.60 ± 0.88% for MAS50, without statistically significant differences, but, in the case of MAS30, the BMD value increased to 28.91 ± 0.86%, with statistical significance (*p* = 0.05) with respect to the other groups. At 60 days, BMD measured only near the periphery of the defect was 39.25 ± 1.28% for the control group and 36 ± 1.72% for MAS30, with the difference that the mineral tissue was more homogeneously distributed in the defect and onto the scaffold. By comparison, the values of the BDM percentage for MAS0 (21. 66 ± 1.73) and MAS50 (27.50 ± 0.94) were low. At 90 days, the lowest value of BMD was for MAS0, 22.12 ± 1.95, followed for MAS50 with a value of 31.23% ± 1.97. However, for MAS30, the BMD continued to increase homogeneously over the whole defect area with a value of 43.39 ± 1.69, in contrast to the control group, which presented a higher degree of mineralization (50.83% ± 1.37) but only in specific areas of the defect, with a statistically significant difference of *p* = 0.0054.

Figure 3F shows the analysis of the total regenerated volume of mineralized tissue deposited in the defect area, as calculated after 90 days. To obtain the value of the total volume, 280 image slices were measured in high-resolution microtomography, calculating the percentage of regeneration to the original size of the defect. For the NT control group, the percentage of regeneration was 3.43 ± 0.35%, which corresponds to the clusters formed near the edges at the periphery of the defect. For MAS0 (6.11 ± 7.54), the percentage of regeneration was statistically similar to that of the NT group. Moreover, for MAS50, the percentage of the total volume of the regenerated mineral was 62.17 ± 9.53% and was statistically different from the NT and MAS0 groups (*p* = 0.0033). Finally, for the group corresponding to MAS30, the total volume of the regenerated area was about 90 ± 5.88%, being statistically significant with regard to the other three groups (*p* = 0.020). This suggests that the MAS scaffold composite material itself could induce the mineralization of the defect area. This biological response is in agreement with the properties of interconnected porosity, which would maximize bone in-growth, leading to osteointegration and strengthening graft fixation due to a larger surface area and more directional in-growth of bone. The osteointegration response of MAS composite scaffold suggests that the material serves as a template for bone-forming cells regulating cell adhesion, migration, growth, and the formation of new mineral tissue related to the presence of HA particles, which serve as nucleation sites and release ions that could serve as stimulatory cues for cell proliferation and cell differentiation, actively induces bone regeneration (Veis and Dorvee, 2013; Gariboldi and Best, 2015; Lotsari et al., 2018).

Upon completing the experimental duration of 90 days, the animals underwent euthanasia so that the quantity and quality of the neo-formed tissue in the area of the defect could be observed by histological analysis. Based on the results, it can be seen that in all of the groups, the defect borders remain well-defined, with a very particular response during the time of the *in vivo* model to the presence or absence of the scaffold in the area of the defect (Supplementary Figure S3).

**FIGURE 4:**
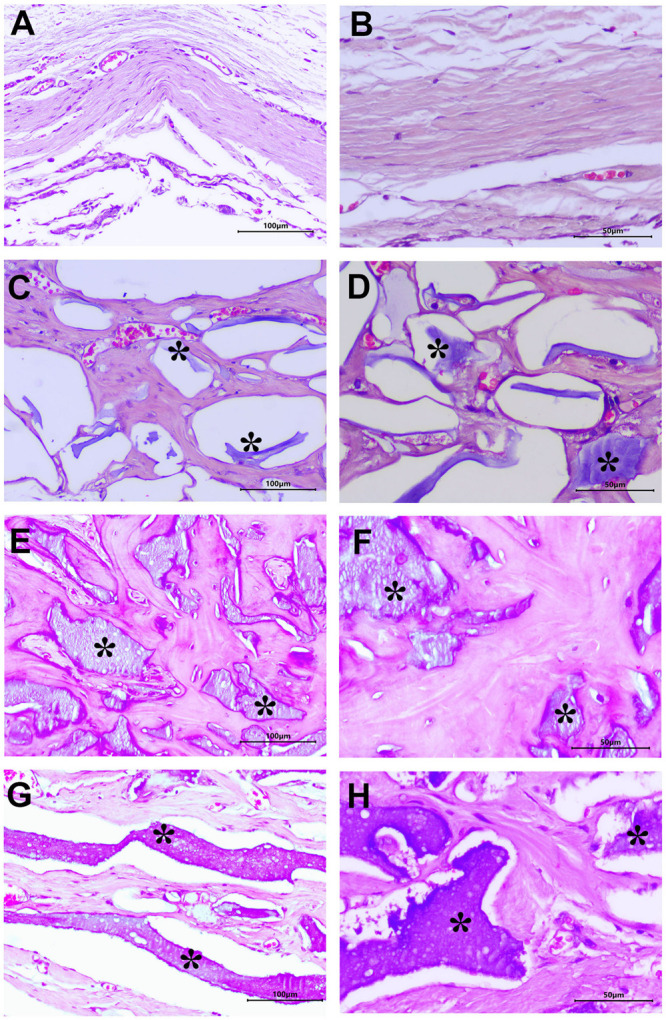
Photomicrographs of histological sections stained with H&E. The images are representative of the different groups at 90 days. **(A,B)** No treatment (NT). **(C,D)** MAS0. **(E,F)** MAS30. **(G,H)** MAS50. All images are shown at 20× and 40×, respectively. The asterisks correspond to the MAS scaffolds.

In the NT control group, the presence of well-organized dense fibrous connective tissue with a proliferation of blood vessels along the defect and small areas of mineralized tissue around the edges were observed (Supplementary Figure S3A and Figures 4A,B). For the MAS0 group, a well-defined border could be observed, with small areas of mineral tissue on the scaffold in the defect zone as well as spaces of different sizes (Supplementary Figure S3B). Moreover, it could be observed to be basophilic and in the form of sheets of different densities and sizes surrounded by partitions of vascularized connective tissue with active fibroblasts. It is important to notice that there were no signs of inflammatory or infiltrate response (Figures 4C,D). The MAS30 group showed a defined border to the defect area, along with multiple zones of basophilic material and *de novo* bone mineral tissue on the cranial defect (Supplementary Figure S3C). At higher magnification, the material could be seen to be a homogenous distribution of bone mineral tissue of irregular shape and density. Around the bone-like tissue, multiple osteocytes in their lacunae, areas of higher osteoid matrix related to the active osteoblast cells, and blood vessel formation could also be seen (Figures 4E,F). For the MAS50 group, irregular dense mineral material could be seen to partially fill the calvarial defect (Supplementary Figure S3D). At higher magnification, the material is eosinophilic, and around it, fibroconnective tissue including blood vessels and few bone-like mineral tissue deposit could be observed (Figures 4G,H). Our results indicate that when the MAS scaffold was loaded with HA, there was earlier osteointegration surrounding the immature new bone trabeculae in the graft area, osteocyte cells became embedded in the lacuna, and there was a decrease in inflammatory cells. This suggests an improvement of the bone-like tissue formation and vascularization processes that are necessary for bone formation by improving the osteoconductivity and osteoinductivity. This can be related to the positive effects of the microporosity of the scaffolds, which allows proper cell migration and proliferation for bone-forming cells as well as tissue vascularization and diffusion of nutrients and oxygen (Gariboldi and Best, 2015; Zhang et al., 2016; Chatzipetros et al., 2018). The improvement of the microporous alginate sponge by loading with HA particles, in conjunction with there being no cytotoxic response or signs of edema and an absence of inflammation, infection, or clinical evidence of gas pocket formation around the scaffolds, means that this could represent an innovative strategy for bone implant materials or as an attractive approach to osteochondral defect regeneration because no significant complications were observed immediately after surgery and throughout the time course of the study.

## Conclusion

In this work, the use of SA sponges with an anisotropic architecture was validated *in vivo*. The sponges efficiently supported the *in vitro* response of hDP-MSC, while bioactive HA embedded in the porous matrix promoted the development of a newly formed mineralized matrix at an *in vivo* calvaria defect. *In vivo* studies confirmed that the presence of the HA phase induces the formation of preferentially oriented pore domains that promote a more ordered spatial organization of the bone ECM architecture, thus supporting a more efficient restoration of the bone defect for long-term clinical trials. In particular, MAS30 delivered the best results in terms of bone-like tissue growth, as confirmed by μCT, BMD, and histological studies, probably due to its more homogeneous distribution and limited tendency to form clusters that are less recognized by cells. Hence, all of the *in vivo* results suggested that these are promising devices for supporting the regeneration of hard tissues such as bone and the mineralized compartment of the osteochondral defect.

## Data Availability Statement

All datasets generated for this study are included in the article/Supplementary Material.

## Ethics Statement

The animal study was reviewed and approved by the Internal Committee for the Care and Use of Laboratory Animals of the Faculty of Medicine, UNAM, with approved protocol number 027-CIC-2019.

## Author Contributions

IC-M, RA, and VG were involved in the fabrication of sponges and their physicochemical characterization. JS-B, FS-O, MA-P, PG-A, IC-M, and LM contributed substantially to the biological assessment experiments and the animal experiments, and performed most of the acquisition, analysis, and interpretation of *in vitro* and *in vivo* data. MA-P, LA, and VG prepared and revised the draft critically for important intellectual content. All authors contributed to the article and approved the submitted version.

## Conflict of Interest

The authors declare that the research was conducted in the absence of any commercial or financial relationships that could be construed as a potential conflict of interest.
