# Supplementary material for: In vivo Regeneration of Mineralized Bone Tissue in Anisotropic Biomimetic Sponges
Source: Front Bioeng Biotechnol. 2020 Jul 7;8:587. doi: 10.3389/fbioe.2020.00587 (PMC7381345; doi:10.3389/fbioe.2020.00587)
Supplement: Supplementary file 1 [file Data_Sheet_1.docx]

**Supplementary data**


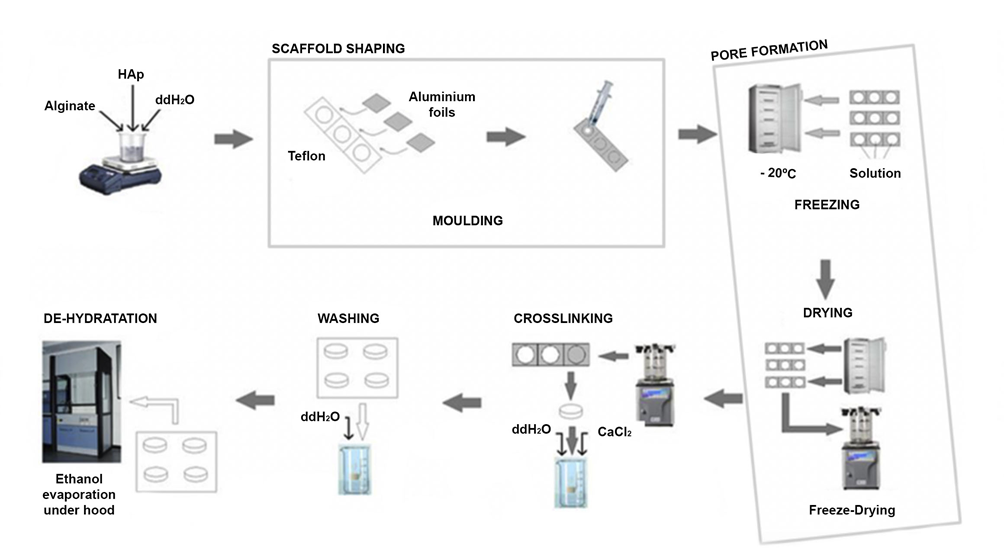


Figure S1: Step by step preparation of alginate porous scaffolds (MAS scaffold): The first step consists of the preparation of a polymeric solution by dispersing (5% wt/v) sodium alginate (SA) extracted from brown algae (viscosity 250 CPps or 20000-40000 CPps - Sigma Aldrich. Milan, Italy) in bidistilled water (ddH_2_O). Then, hydroxyapatite particles (HAp) from Plasma (theoretical density 3.16 g = cm3 - Biotal, Tideswell, UK) - spherical shape and bimodal size distribution (Frange ¼ 0.4–11 mm, F0.5¼ 4.02 mm) [23] - were added, respectively 30% or 50% volume with respect to the polymer weight. Solutions were left under magnetic stirring for about 24 h at room temperature until complete dissolution. Then, 0.4 ml of solution was arranged in highly thermally insulated TEFLON molds and subsequently placed in the freezer at a temperature of -20 °C for about 2 h to trigger phase separation mechanisms, i.e., nucleation and the growth. The freeze-drying step was assessed for 24 h to remove ice crystals via sublimation in order to form the macropores. After the removal from the mold, samples were immersed in a solution containing 1.1% wt/v of calcium chloride (CaCl_2_, Sigma Aldrich, Italy) for 1 h so that the crosslinking of the alginate polymer chains took place. Subsequently, the samples were washed in ddH_2_O to eliminate residual salts and waste. After a brief drying step under a hood (1 h) to remove the surface water, composite sponges were sequentially dipped in four ethanol/water solutions (20/80 v/v, 50/50 v/v, 80/20 v/v, and 100/0 v/v) to completely remove water from the polymeric network and to prevent uncontrolled volumetric shrinkage of the scaffold. Finally, disc-shaped samples 10 mm in diameter and 2 mm in height were used for *in vivo* studies. For this use, composite alginate sponges were washed with sterile bi-distilled water six times and then immersed in pure ethanol for 1 h for mild sterilization.


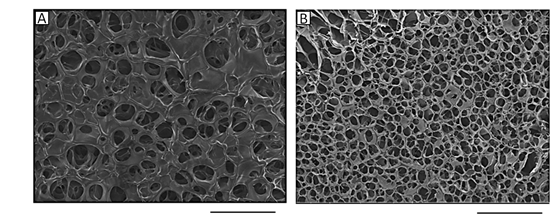


Figure S2: Comparative SEM analysis of porous sponges made of alginate with different molecular weight (scale bar: 250 micron): A) low molecular weight (viscosity 250 CPs) and B) high molecular weight (viscosity 20000-40000 CPs). SEM images show an increase in the average pore size – from 45±11 to 96±14 microns as calculated via image analysis - moving from high to low viscosity alginates and improvements in terms of degree of porosity and pore interconnection. As expected, the mechanism of phase separation is strongly influenced by the peculiar properties of the polymer. In particular, ice crystals tend to be smaller in the presence of high molecular weight solution where the nucleation mechanisms are activated but the kinetics of the phase growth process are constrained in order to obtain pores large enough to promote cell colonization. Samples A were selected for the biological studies.


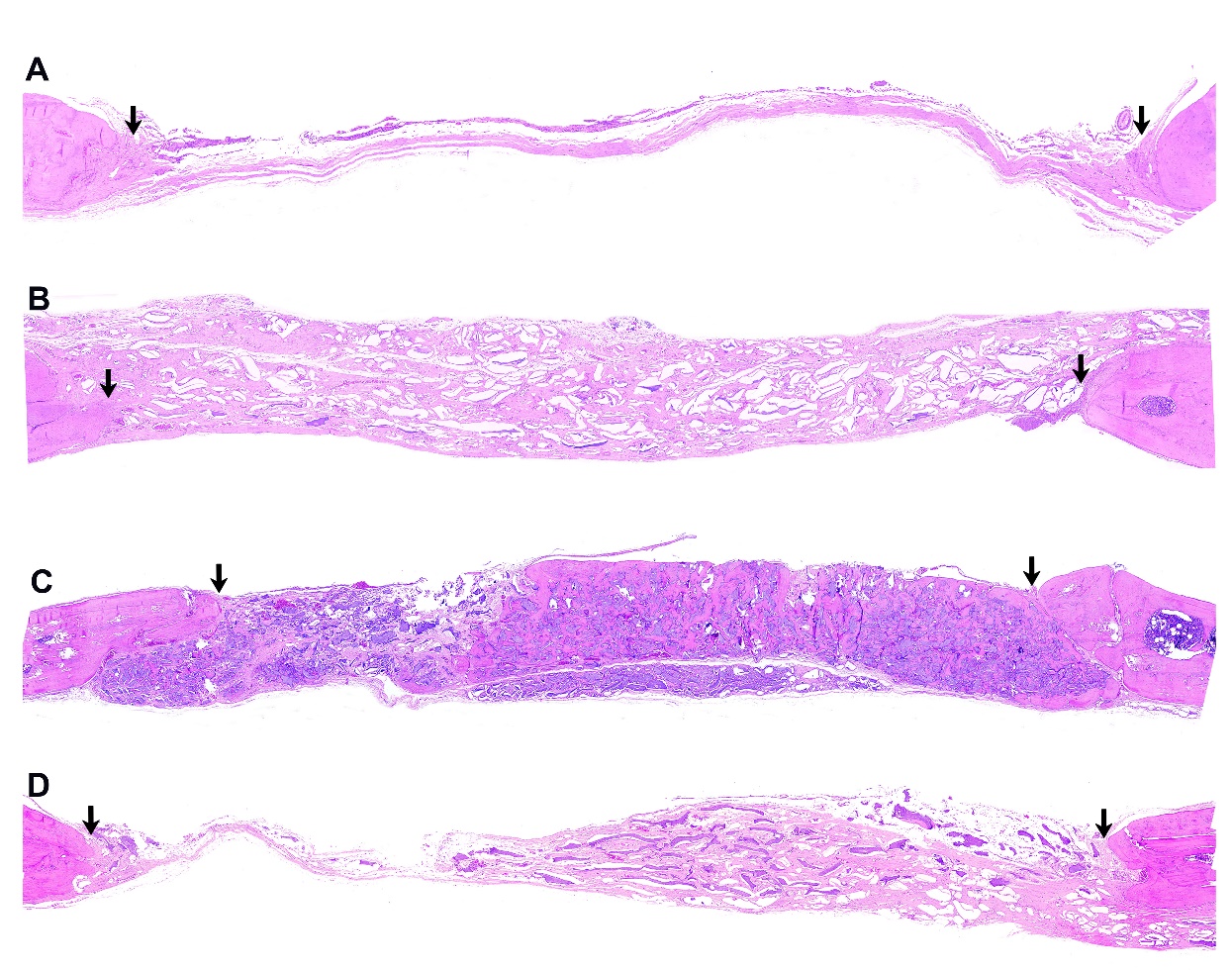


Figure S3. Representative histological images of the in vivo studies 90 days after surgery. A) Non-treated (NT) group, B) MAS0, C) MAS30, and D) MAS50 group. The arrows indicate the edges of the bone defect. The results suggest that MAS30 enhanced bone regeneration compared to the MAS 50 and MAS0 group. However, bone regeneration on the MAS composite scaffold could be related to the presence of HA particles crosslinked to the alginate polymer. The methodological procedures of the histological images consisted of an excisional biopsy of the defect area performed with a safety margin of 10 mm. Then, the sample was thoroughly washed and placed in 10% formalin for 24 hours for fixation. Subsequently, the samples were washed for 2 hours under running water and placed in an Evans and Krajian solution for demineralization. Finally, for the histological process, the samples were put in a Histokinnet, dehydrated in increased alcohol concentrations of 50%, 70%, 90%, and 100%, and embedded in paraffin blocks. Serial sections 5 micrometers thick were made and stained with hematoxylin and eosin (H & E).
